# Factory Tsetse Flies Must Behave Like Wild Flies: A Prerequisite for the Sterile Insect Technique

**DOI:** 10.1371/journal.pntd.0000907

**Published:** 2011-02-22

**Authors:** Marc J. B. Vreysen, Khalfan M. Saleh, Renaud Lancelot, Jérémy Bouyer

**Affiliations:** 1 Insect Pest Control Laboratory, Joint FAO/IAEA Programme of Nuclear Techniques in Food and Agriculture, Vienna, Austria; 2 Ministry of Agriculture, Natural Resources and Environment, Zanzibar, Tanzania; 3 Cirad, UMR Contrôle des maladies animales exotiques et émergentes, Campus International de Baillarguet, Montpellier, France; National Institute of Allergy and Infectious Diseases, United States of America

Tsetse flies are the vectors of human and animal African trypanosomoses, the former a major neglected disease, and the latter considered among the greatest constraints to livestock production in sub-Saharan Africa. To date, the disease is mainly contained through the prophylactic and curative treatment of livestock with trypanocidal drugs, which is not sustainable. The removal of the vector, the tsetse fly, would be the most efficient way of managing these diseases. A number of efficient tsetse control tactics are available that can be combined and applied following area-wide integrated pest management (AW-IPM) principles [1]. The concept entails (1) the integration of various control tactics, preferably combining those methods that are effective at high population densities with those that are effective at low population densities to obtain maximal efficiency, and (2) the control effort is directed against an entire tsetse population within a delimited area. This is particularly relevant in case eradication is the strategy of choice. Genetic control tactics such as the sterile insect technique (SIT) show great potential for integration in such AW-IPM programmes because they are very efficient for controlling low-density populations, which is not the case for most other techniques. Sterile male insects are reared and, after sterilization with ionizing radiation, sequentially released in large quantities to outnumber the wild male flies. A mating of a sterile male with a virgin wild female fly results in no offspring. Recently, transgenic and paratransgenic techniques have been proposed to sterilize male insects or to make strains refractory to disease parasites in the case of vectors [2]–[4]. However, to ensure the success of these control methods, factory-reared tsetse flies must be competitive with their wild counterparts and must exhibit a similar behaviour in a natural environment.

The SIT as part of an AW-IPM approach is a robust technique, which has proven to be very efficient in eradicating, suppressing, or containing dipteran pests such as *Cochliomyia hominivorax* (New World screwworm) in Central America, Mexico, the United States, and Libya [5], [6], *Ceratitis capitata* (Mediterranean fruit fly) in Argentina, Chile, Israel, Mexico, Peru, Spain, and the US, *Bactrocera cucurbitae* (melon fly) in the Okinawa archipelago of Japan, and lepidopteran pests such as *Cydia pomonella* (codling moth) in Canada, Australian painted apple moth (*Teia anartoides*) in New Zealand, and *Cactoblastic cactorum* (cactus moth) and *Pectinophora gossypiella* (pink bollworm) in Mexico and the US [1]. Similarly, the SIT has been successfully integrated with other control tactics against several tsetse species, i.e., with aerial spraying of insecticides against *Glossina morsitans morsitans* in Tanzania, with insecticide-impregnated targets and traps against *Glossina palpalis gambiensis* and *Glossina tachinoides* in Burkina Faso and *G. palpalis palpalis* in Nigeria, and with the live-bait technique against *Glossina austeni* on Unguja Island (Zanzibar). These programmes showed that the SIT against tsetse is feasible, but with the exception of the programme on Unguja Island [7], they proved to be unsustainable.

Some have thus questioned whether competitive sterile male tsetse flies can be produced [8], especially since learning mechanisms like site- [9] or host-fidelity [10] might influence their behaviour and prevent them from feeding efficiently on wild hosts after being reared on artificial membranes in a laboratory environment. Understanding all the factors that contributed to the success on Unguja Island would be useful for future eradication programmes.

Earlier work has already demonstrated that laboratory reared and released sterile tsetse flies were able to feed on wild hosts: (1) recaptured sterile male flies often had residues of blood meals in their digestive tract, (2) a mean lifespan in nature of 11–17 days [9], which was similar to captured, marked, and released wild males [11], and (3) released sterile male tsetse that received too low a dose of isomethamidium chloride in their blood meal before release were found infected with trypanosomes after release in a natural environment [12], which would have been impossible in the absence of a blood meal on wild hosts.

Adequate survival, dispersal, dispersion, mobility, and mating compatibility are critical factors influencing sexual competitiveness of the released sterile male flies [13]. An analysis of the data collected during the AW-IPM programme against *G. austeni* on the Island of Unguja indicated that the sterile males did not disperse randomly but showed the same spatial distribution as their wild counterparts. The most detailed data sets were available from the primary Jozani Forest Reserve (now part of the Jozani-Chwaka Bay National Park) (6°15′S and 39°25′E), where the vegetation was very homogeneous and where >300 sterile male flies were released per week per km^2^. All sterile male flies were marked with fluorescent dye, irradiated with 120 Gy, packaged in carton release containers, and released by air at an altitude of 250 m. The release of the sterile male flies by air ensured their random distribution on the island. The release cartons were dropped at very regular intervals and opened upon contact with the airstream that forced the flies out of the carton box. By the time the flies reached the vegetation, they were not clustered anymore but occupied a certain area of surface in a random way. The flies were sampled with 12 royal blue–white leg panels made sticky with the non-setting adhesive Temoocid. Data sets from week 40, 1994 (start of operational release) to week 26, 1995 were used for the analysis. Thereafter, the number of wild *G. austeni* flies was too low to be meaningful for the analysis. The sticky panels were checked every weekday. The vegetation around each sticky panel was cleared within a radius of 3 m to ensure homogeneous trap efficiency. After collection, all flies were transferred to the laboratory at the Zanzibar Commission of Agriculture and Livestock to examine the head capsules for fluorescence under a UV microscope to distinguish sterile from wild flies.

The main purpose of the statistical analysis was to assess similarities or differences in the spatial pattern of apparent densities of wild and sterile male flies using capture records from the sticky panels. Firstly, we tested the existence of a spatial trend in wild male counts (after log transformation), and we subtracted this trend from log-counts before investigating the independence of trap locations and wild male fly abundance. This was achieved with a Monte Carlo test for marked point processes [14]: the point process being the set of trap locations, and the marks being the wild male fly counts. Secondly, we used a χ^2^ test to assess the spatial heterogeneity in wild male fly abundance, and correlation tests to assess the independence of wild males and females, and sterile males.

To plot the data, we transformed fly counts into standardized contributions. For each fly category *i* (wild male or female, sterile male) and trap *j* (*j* = 1,… *J*), each observed trap count *n_i,j_* was divided by the total observed count *N_i_* for this fly category to give the observed relative contribution of each trap *o_i,j_* = *n_i,j_*/*N_i_*. The expected relative contribution of trap *j* under the assumption of homogeneous spatial distribution (*e_i,j_* = 1/*J*) was then subtracted to *o_i,j_* and the result was divided by *e_i,j_*, thus providing the standardized contribution *c_i,j_* = (*o_i,j_*−*e_i,j_*)/*e_i,j_* = *J n_i,j_*/*N_i_*−1.

**Figure 1:**
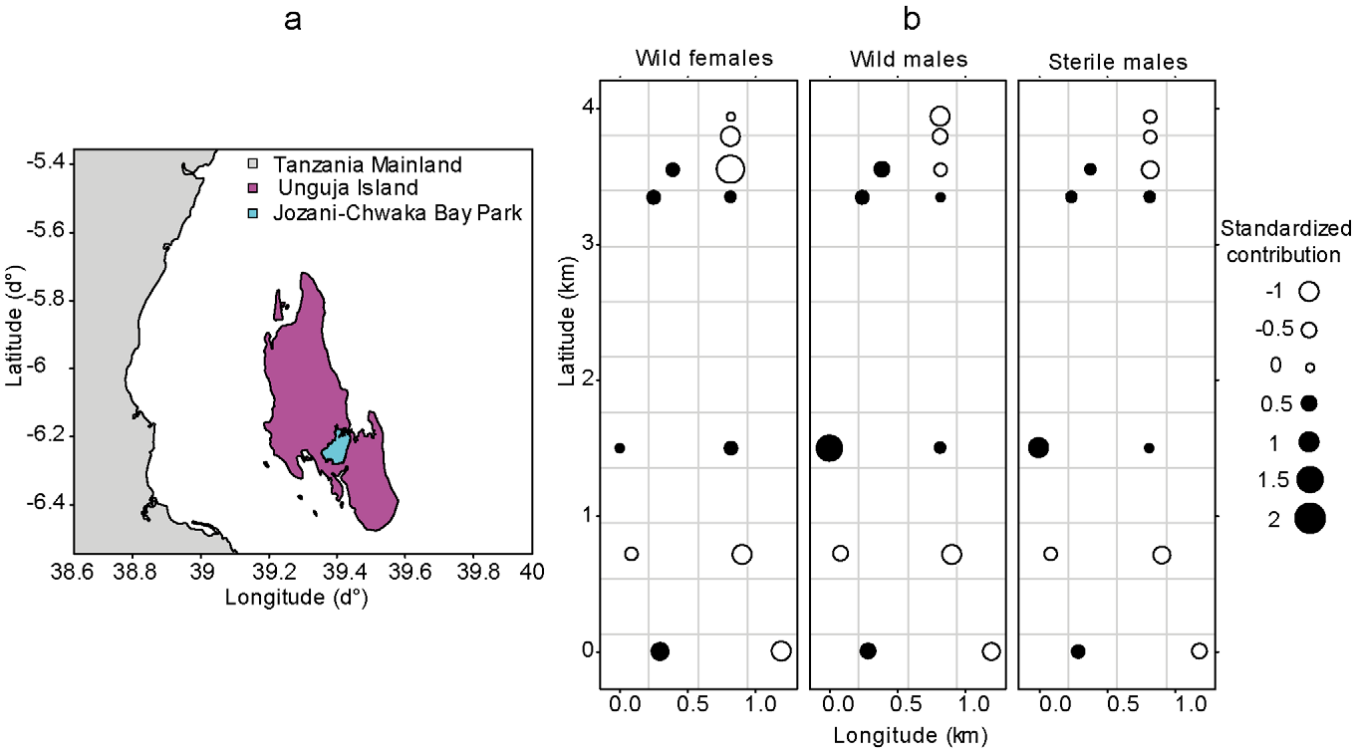
Description of the trapping system. (a) Unguja Island, and (b) spatial distribution (standardized abundance) of wild and sterile *Glossina austeni* as sampled with 12 sticky panel traps in the Jozani-Chwaka Bay National Park. See text for explanations on abundance standardization. Longitude and latitude are expressed in km, with the origin at the bottom left corner of the virtual rectangular box bounding trap locations.

A total of 422 wild female, 679 wild male, and 3,318 sterile male *G. austeni* were trapped in the 12 monitoring sites over this 10-month period (Figure 1). Wild male fly trap catches were higher in the northern part of the forest (linear trend, *R* ^2^ = 0.38, *p* = 0.03). A similar trend was observed for wild female (*R* ^2^ = 0.72, *p* = 5.10^−4^) and sterile male flies (*R* ^2^ = 0.46, *p* = 0.02). These spatial trends were removed from the data sets for further analyses. The point marked process analysis showed that wild male fly counts were independent from trap locations (Monte Carlo test, *p*>0.05), i.e., no interaction was detected between trap locations and fly counts.

**Figure 2:**
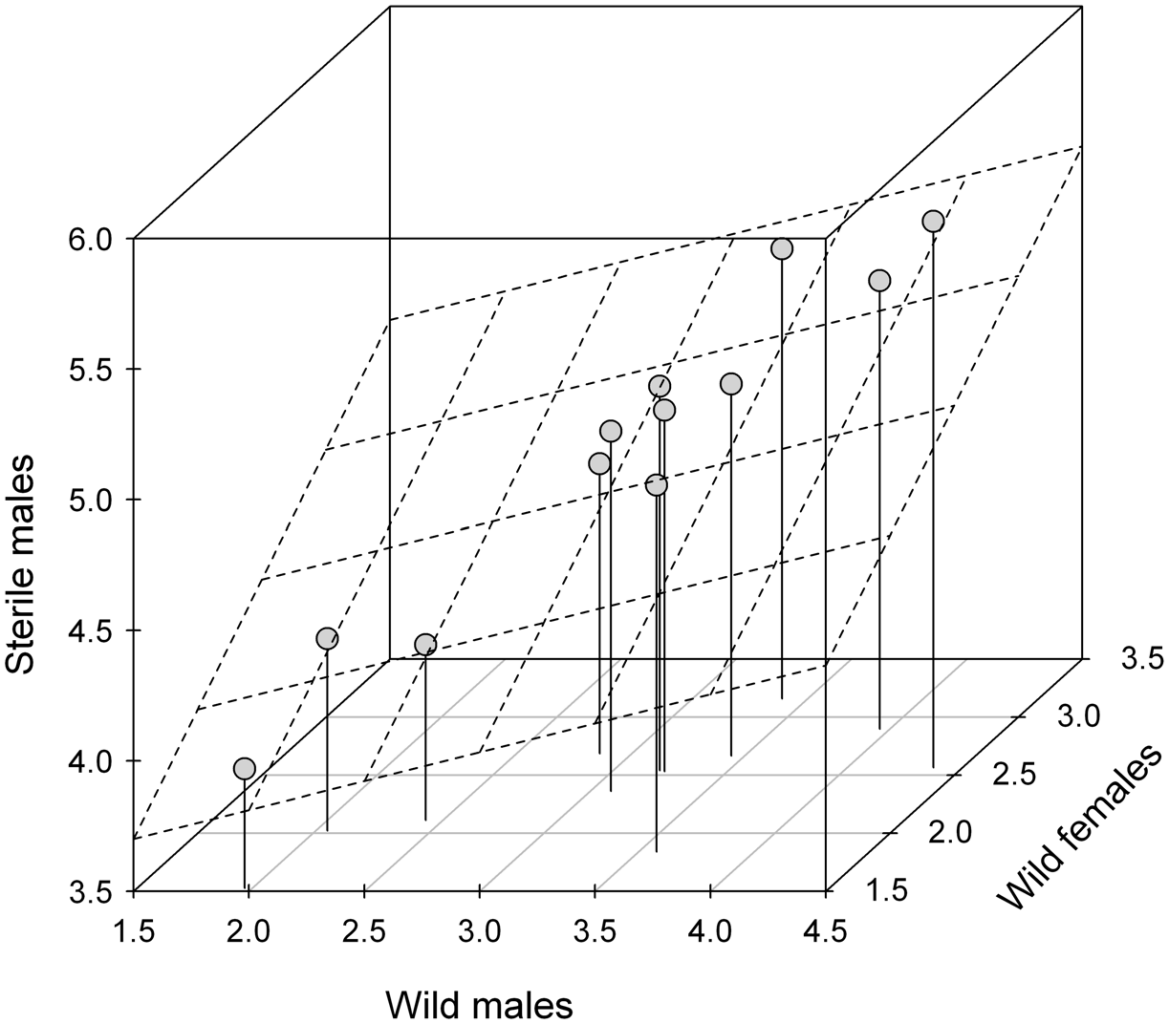
Joint distribution of wild and sterile *Glossina austeni* in the Jozani-Chwaka Bay National Park. Data are log counts from which spatial trends have been removed. A regression plane (dashed lines) of sterile male fly counts versus wild fly counts (males and females) was added to the scatter plot (*R* ^2^ = 0.95, *p*<10^−4^).

Although sterile male flies were uniformly dispersed by light aircraft over this forest, their spatial distribution, as evidenced by trapping counts—with spatial trend removed—was highly heterogeneous (χ^2^ = 302, df = 11, *p*<10^−4^). The distributions of wild male and female flies were also heterogeneous (χ^2^ = 28, df = 11, *p* = 0.004; χ^2^ = 165, df = 11, *p*<10^−4^). The joint distribution of these de-trended counts is shown in Figure 2. The distribution of sterile and wild male flies was highly correlated (*r* = 0.96, *p*<10^−4^), as was the distribution of sterile male and wild female flies (*r* = 0.72, *p* = 0.01). The correlation between the distributions of wild male and female flies was lower, but still significant (*r* = 0.61, *p* = 0.04). This weaker correlation might be related to different, sex-specific preferences in fly habitat.

The linear spatial trend showed that the observed heterogeneous distribution among trap positions cannot be explained by differences in trap efficiency, but by an aggregation in certain preferred sites. Barclay [15] has shown the importance of insect aggregation in pest control, especially when using the SIT or any other genetic control method. Even in this fairly homogeneous primary forest habitat on Unguja Island, the distribution of wild *G. austeni* was heterogeneous and thus aggregated, as was observed in South Africa [16]. The ability of sterile males to aggregate (and thus locate) those areas preferred by the wild males is of primary importance to ensure adequate sterile-to-wild male ratios everywhere and was therefore an important factor contributing to the success of the programme. It would be important to reconfirm this observation in other programmes that have a sterile insect component where it should be included as a quality control measure. In addition, the present data suggest that tsetse fly dispersal cannot be solely considered as a homogeneous diffusion process, as often assumed [9], [17]. It confirms that mass-reared and gamma-sterilized male *G. austeni* were able to respond to environmental cues and to aggregate in the preferred sites of the wild population. Their dispersal behaviour was therefore similar to that of wild flies, which confirms that tsetse flies are very good candidates for genetic control.
